# Special feature: measuring components of ecological resilience in long-term ecological datasets

**DOI:** 10.1098/rsbl.2020.0881

**Published:** 2021-01-27

**Authors:** Alistair W. R. Seddon

**Affiliations:** 1 Department of Biological Sciences, University of Bergen, Bergen, Norway; 2 Bjerknes Centre for Climate Research, Bergen, Norway

**Keywords:** palaeoecology, ecological resilience, long-term ecology, temporal dynamics, recovery rate

## Abstract

Ecological resilience has become a focal concept in ecosystem management. Palaeoecological records (i.e. the sub-fossil remains preserved in sediments) are useful archives to address ecological resilience since they can be used to reconstruct long-term temporal variations in ecosystem properties. The special feature presented here includes nine new papers from members and associates of the PAGES EcoRe3 community. The papers build on previous work in palaeoecology to investigate, identify and compare components of ecosystem resilience on centennial to millennial timescales. There are four key messages that can be summarized from the findings of papers within the special feature: (i) multi-proxy studies reveal insights into the presence and mechanisms of alternative states; (ii) transitions between alternative states may not necessarily be abrupt; (iii) components of ecological resilience can be identified in long-term ecological data and (iv) the palaeoecological record can also provide insights into factors influencing the resilience of ecosystem functioning. Overall, these papers demonstrate the importance of using long-term ecological records for addressing questions related to the theoretical framework provided by ecological resilience.

## 1. Investigations into ecological resilience

Ecological resilience has become a focal concept in ecosystem management [1] and underlies several of the United Nations Sustainable Development Goals [2]. Studies that investigate the patterns and underlying drivers behind ecosystem resilience have become common across all areas of ecology, from laboratory manipulations [3], empirical observations of natural and experimental communities [4], to global-scale analyses of satellite data [5]. Across these studies, resilience is commonly conceptualized in two ways.

*Ecological resilience* describes the capability of a system to tolerate a disturbance and remain within the same functional and structural state [6,7]. Central to this concept is the basin of attraction, which describes the location of the stable and unstable attractors in the system in relation to environmental forcing(s). Under this model, the shape of this basin of attraction determines the overall system resilience. Critical transitions occur when resilience is low and when positive feedbacks cause large and sudden changes between ecosystem states following small external or internal perturbations [8]. Thus, global change ecologists are concerned with identifying the presence of alternative equilibria within ecological systems, and in identifying the factors that might lead to high ecological resilience in order to limit the potential for major ecosystem transitions [9].

*Engineering resilience* describes the rate of recovery of a system to a perturbation [10]. This concept has most commonly been applied across field, laboratory and experimental studies through measurement of the rate of the recovery to an equilibrium point [4]. A link between engineering and ecological resilience has been identified based on theoretical and experimental evidence that indicates that the rate of recovery of a system decreases (i.e. the *ecological* resilience declines) as a system approaches a critical threshold [11]. Thus, the recovery rate has been suggested to act as a quantitative proxy of ecological resilience and may serve as an early-warning indicator for an impending regime shift [12]. More recently, multiple components of resilience have been used to describe properties of the underling stability landscape, including consideration of recovery rate, resistance (the amount of change experienced by a system followed by a disturbance), latitude (the width of an underlying stability landscape) and precariousness (the probability of a threshold shift) [13].

## 2. Ecological resilience and long-term ecology

To fully understand the factors that might lead to reduced or enhanced ecosystem resilience, it is necessary to appreciate the sequence of historical processes that have influenced the ecosystem up to the present day [14]. Palaeoecological records (i.e. sub-fossil remains preserved in sediments) are useful archives in this regard since they can be used to reconstruct temporal variations in ecosystem properties. Furthermore, palaeoecological records can provide information beyond that offered by traditional ecological studies, which are typically based on measurements representing timescales of years to decades [14–16]. Indeed, palaeoecologists have attempted to identify ecological phenomena typical to the processes related to ecological resilience through: identifying regime shifts and critical transitions [17]; determining the underlying factors leading to abrupt changes in ecosystems [18]; identifying early-warning indicators prior to ecosystem collapse [19] and quantifying and comparing recovery rates within and between geographic regions and ecosystems [20].

The PAGES (Past Global Changes) EcoRe3 working group (http://pastglobalchanges.org/science/wg/ecore3/intro) was established to explore methods and approaches for investigating components of ecological resilience in long-term ecological data. This special feature includes nine new papers from members and associates of this community [21] and builds on the previous work to investigate, identify and compare components of ecosystem resilience within and between ecological systems. Taken together, there are four common themes across the papers in this special feature.

### (a) Multi-proxy studies reveal insights into the presence and mechanisms of alternative states

The majority of studies in this special feature involve the analysis of multiple proxies, so that both the environmental driver(s) and the ecosystem response can be reconstructed simultaneously. For example, Morris *et al*. [21] develop a long-term record of fire history from a lake site surrounding an aspen forest in western North America. Based on contemporary ecological understanding, North American quaking aspen (*Populus tremuloides*) forests can exist in two states: stable (monotypic) or seral (subject to periodic invasion by subalpine fir, *Abies lasiocarpa*). Direct observations of fire history are difficult to observe in these ecosystems, and so the relative importance of fire in determining the emergence of seral or stable quaking aspen states is challenging to establish. To address this issue, the authors use a combination of charcoal and pollen data to show that the quaking aspen forest transitioned from a seral to a stable state as a result of high-severity fires approximately 1500 years before present. The charcoal record for the past 500 years shows lower incidences of fire relative to past periods, but pollen data from this site indicates that forest shows evidence of moving back towards a seral state today.

Similarly, Gil-Romera *et al*. [22] use a combination of reconstructions of vegetation and fire activity to identify drivers of vegetation shifts in sub-tropical-montane systems in Ethiopia. Current knowledge of *Erica*–fire relationships in the Ethiopian sub-tropical-montane ecosystem have generally been based on studies of modern vegetation, and although likely present for millennia, fire has been identified as a negative driver in terms of erosion, carbon storage and vegetation resilience in these ecosystems [22]. In their paper, Gil-Romera *et al*. use multi-proxy evidence to show that fire occurrence was in operation throughout the Lateglacial and Holocene. Additionally, they use a statistical approach to show that pollen-accumulation rates of Ericaceae vegetation lagged behind charcoal-accumulation rates (with positive correlations between 550- and 10-year lags). Thus, they argue that a positive feedback between Ericaceae vegetation and fire was in operation throughout the Lateglacial and Holocene and interpret the ericaceous belt of the Ethiopian mountains as a fire-resilient ecosystem.

Finally, Xu *et al*. [23] use diatom assemblages and geochemical evidence to determine the underlying dynamics of lake regimes shifts from two lakes in China. Regime shifts in diatom assemblages occurred in both lakes, one in the 1950s and another in 1970s. However, the authors show that the mechanisms of regime shifts in these two lakes were different. While one lake experienced a transition as a result of extrinsic changes in the basin, in the second lake, ongoing nutrient loading influenced ecosystem processes and drove the lake to an alternative stable state. This latter example potentially presents an example of a critical transition after a loss of resilience. Taken together, these three papers demonstrate the power of long-term proxy information to both detect and attribute regime shifts, in addition to behaviour related to ecological resilience, across a range of systems.

### (b) Transitions between alternative states may not necessarily be abrupt

Abrupt ecosystem change is often considered to be a hallmark of a regime shifts between alternative states. For example, the spatial discontinuities between forest and savannah ecosystems observed in present-day sub-tropical and tropical ecosystems are thought to be a result of fire-feedbacks which can suppress (i.e. forests) or promote (i.e. savannahs) fires. Transitions between alternative states over time are likewise also often assumed to be abrupt. However, there are few examples demonstrating the temporal transition from forest to savannahs (or vice-versa) in empirical datasets. Using a multi-proxy approach, which included the collation of archaeological evidence to determine the extent of historic human impact in the site, Aleman *et al*. [24] investigated the dynamics of one such regime shift between forest and savannahs using a sediment record from southern Congo, Africa. They showed that the transition from closed canopy forest to open savannah took at least 500 years, and the transition to a full savannah took even longer. Although fire activity was increasing, local forest appeared to remain resilient in the face of increasingly frequent fires for at least 500 years. Thus, even in systems where abrupt and rapid transitions are assumed to be common, palaeoecological evidence shows that transitions can occur gradually, over timescales that may be more difficult to perceive from standard human perspectives [25]. Such findings are interesting in the light of a recent meta-analysis that found no evidence of threshold type behaviour using empirical datasets [26].

### (c) Components of ecological resilience can be identified in long-term ecological data

Many empirical studies in the ecological literature propose the use of statistical methods to identify components of ecological resilience [13]. Two papers in this special issue provide examples of attempts to determine similar metrics using long-term palaeoecological data under this theme. Adolf *et al*. [27] use 30 long-term, high-resolution palaeoecological records from Mexico, Central and South America to compare recovery rates in tropical ecosystems. They investigate recovery rates of arboreal-pollen taxa by identifying when tropical forests experienced large declines in pollen abundance and then measured the return time of this change to a statistically defined equilibrium point. They then use these data to test two hypotheses related to the underlying causes of resilience: that increased biodiversity will speed up the rates of recovery in tropical systems, and that abiotic factors that are location specific can result in similar patterns of resilience today as compared to the past.

Similarly, Calder & Shuman [28] develop a statistical framework for examining resilience in palaeoecological records against the backdrop of a non-stationary climate change, as determined through analysis of pollen compositional changes, stable isotope records and fossil charcoal. They use a statistical model to predict the equilibrium pollen percentage values for a given time point and then estimate vegetation resistance (the magnitude of change) and vegetation recovery (time required to return) to these predicted equilibrium values. Their approach recognizes that, because climatic and environmental conditions can change over time, shifting baselines will mean that the equilibrium state will also shift. This is an important concept which is often neglected, since equilibria are unlikely to be stable over the time periods (i.e. centuries to millennia) considered by palaeoecological sequences.

### (d) The palaeoecological record can also provide insights into factors influencing the resilience of ecosystem functioning

Changes in community composition of both aquatic and terrestrial systems have been a major focus in resilience studies in palaeoecology, but recently there has been a move to better understand the long-term dynamics of biogeochemical cycling and ecosystem functioning. In this special feature, Chipman & Hu [29] combine fire, vegetation and biogeochemical analyses to investigate the impacts of disturbance on nutrient cycling within an Alaskan terrestrial lake catchment. In their study, increasing lake sediment *δ*^15^N values over the Holocene reflect the development of *Picea mariana* dominated forests, which cause soils to become increasingly enriched with the heavier ^15^N isotope as *P. mariana* fix organic N via ectomycorrhizae. Short-term deviations in *δ*^15^N values were also observed following evidence of catchment level fires, but the sediment *δ*^15^N values returned to pre-fire values within 50 years. This leads the authors to conclude that N cycling within this watershed was resilient to fire disturbance over the late Holocene. Sediment *δ*^15^N records are also used by Bonsall *et al*. [30] in this issue, who develop a hierarchical model-selection approach to test for feedbacks that might exist between soil mycorrhiza and the plants that control plant–nutrient interactions.

Finally, one paper from this special issue indicates that abrupt changes across multiple ecosystem components do not necessarily occur simultaneously. Lamentowicz *et al*. [31] use a combination of testate amoebae and *Sphagnum* macrofossils to reconstruct the changes in sphagnum-community composition of several peat cores in relation to water-table depth. Their data indicate an ecological threshold in relation to water-table depth in these peatland systems, across which the community composition of *Sphagnum* changes abruptly. However, the functional diversity in the *Sphagnum*-community traits did not change over this time period. Thus, while *Sphagnum*-community composition was subject to ecosystem shifts in response to hydrological variations, major *Sphagnum* ecosystem functioning remained stable across the inferred regime shifts.

## 3. Synthesis

The long-term perspectives offered by the papers in this special feature reveal multiple instances of ecosystem reorganization in response to extrinsic environmental forcing in the past. Indeed, while a recent meta-analysis found no evidence of threshold type behaviour in ecological systems along temperature gradients [25] and suggested that ‘global change biology needs to abandon the general expectation that system properties allow defining thresholds as a way to manage nature under global change’, the studies presented here demonstrate the importance of taking a long-term perspective for understanding ecosystem change in response to environmental drivers. Overall, these papers demonstrate that multi-proxy studies are an important resource for global change ecology. Sediments provide a unique opportunity to better understand non-linear ecosystem dynamics, and for gaining novel conceptual and practical insights related to the theoretical framework provided by ecological resilience.

## Data accessibility

This article has no additional data.
